# Low-dose (0.15 mg/kg) versus high-dose (0.6 mg/kg) dexamethasone for pediatric croup: a meta-analysis with trial sequential analysis of RCTs

**DOI:** 10.3389/fped.2026.1925971

**Published:** 2026-08-21

**Authors:** Ayesha Khalid, Susan Flesher, Thaís S. Martins Shehan

**Affiliations:** 1 Joan C. Edwards School of Medicine, Marshall University, Huntington, WV, United States; 2 Universidade Federal Fluminense, Niterói, Brazil

**Keywords:** corticosteroids, croup, dexamethasone, dose comparison, pediatric airway disease, randomized controlled trials, trial sequential analysis

## Abstract

**Introduction:**

Systemic glucocorticoids are the standard treatment for croup. Although dexamethasone 0.6 mg/kg is widely recommended, lower doses may provide similar benefit. To compare oral dexamethasone low dose (0.15 mg/kg) to high dose (0.6 mg/kg) in children with croup.

**Methods:**

We systematically searched PubMed, Embase, and Cochrane Library from inception to November 25, 2025. We restricted our analysis to randomized controlled trials (RCTs) directly comparing low-dose vs high-dose oral dexamethasone in children with croup. Risk ratios (RRs) were calculated for binary outcomes and mean differences (MDs) for continuous outcomes, with 95% confidence intervals (CIs). We performed a random-effect meta-analysis for all outcomes using R software (version 2025.09.2+418).

**Results:**

Five RCTs were included, encompassing 1,058 patients, of whom 50% received low-dose dexamethasone and 50% received standard dose. We found no significant difference between doses for croup severity scores at 1hr, 3hr and 4hr (MD −0.05 points; 95% CI -0.37 to 0.27), (MD 0.00 points; 95% CI -0.42 to 0.42) and (MD −0.03 points; 95% CI -0.19 to 0.12) respectively, hospital admission (RR 1.06; 95% CI 0.58 to 1.94), return to healthcare (RR 1.05; 95% CI 0.67 to 1.64), and need for additional racemic epinephrine (RE) (RR 1.58; 95% CI 0.77 to 3.25).

**Discussion:**

In this meta-analysis, low-dose oral dexamethasone (0.15 mg/kg) was comparable to the traditional 0.6 mg/kg dose in children with mild to moderate croup, with similar clinical outcomes in croup severity scores at 4 hours, hospital admission, return to healthcare, and need for additional RE. However, trial sequential analysis suggests that the current evidence remains underpowered and inconclusive, as the required information size was not reached for key outcomes. The small number of available RCTs limited statistical power to detect modest differences in effect size. Although we sought to evaluate dose equivalence in children with more severe croup (baseline croup score >5), only three trials included this population, and outcome reporting was insufficiently standardized to allow a meaningful subgroup synthesis. These findings should not be extrapolated to children with severe croup, and adequately powered randomized trials are needed before definitive dosing recommendations can be made.

**Systematic Review Registration:**

identifier CRD420251238877.

## Introduction

Croup is one of the most common respiratory illnesses in early childhood (1). Although most cases are mild, croup accounts for nearly 7% of hospitalizations among children under five years (1, 2). It primarily affects infants and toddlers between 6 and 36 months of age, with peak incidence during the second year of life (3). It typically presents after an upper respiratory prodrome and is characterized by a barking cough, inspiratory stridor, hoarseness, and varying degrees of respiratory distress (4, 5).

Glucocorticoids are the main treatment for croup of any severity and are shown to rapidly reduce symptom burden, shorten hospital length of stay, and decrease return visits or readmissions (6). Oral dexamethasone is the preferred corticosteroid; however, the optimal dose remains uncertain. While 0.6 mg/kg continues to be recommended in many guidelines, lower doses, particularly 0.15 mg/kg, may provide similar clinical benefit (7).

In 2023, a Cochrane review synthesized evidence from 45 randomized controlled trials (RCTs) involving 5,888 children, evaluating glucocorticoids across multiple formulations, routes of administration, and dosing strategies (7). The review suggested that dexamethasone 0.15 mg/kg may provide clinical outcomes comparable to the standard 0.6 mg/kg dose (7). However, whether the currently available evidence is sufficient to support routine use of the lower dose, or whether additional randomized trials remain necessary, has not been formally evaluated.

In this context of **high-value care**, as promoted by the American Academy of Pediatrics (AAP) (8), it is important to reassess whether traditional dosing strategies offer a meaningful clinical advantage over lower-dose regimens (9). Although generally well tolerated, even short courses of systemic corticosteroids have been associated with adverse effects, including hyperglycemia, sleep disturbance, and gastrointestinal bleeding (7).

Therefore, we conducted a systematic review and meta-analysis of RCTs comparing oral dexamethasone 0.15 mg/kg and 0.6 mg/kg in children with croup. In addition, we performed trial sequential analysis (TSA) to evaluate the robustness and sufficiency of the accumulated evidence, determine whether the currently available evidence supports reliable conclusions regarding optimal dexamethasone dosing, and establish whether further studies are needed.

## Methods and materials

This systematic review and meta-analysis with TSA were conducted in accordance with Cochrane Collaboration Handbook for Systematic Reviews of Interventions and reported in compliance with Preferred Reporting Items for Systematic Reviews and Meta-Analyses (PRISMA) guidelines (10, 11). The study protocol was registered in the International Prospective Register of Systematic Reviews (PROSPERO) under the ID “CRD420251238877”. Although trial sequential analysis (TSA) was not specified in the original registered protocol, it was subsequently performed to assess the robustness and sufficiency of the accumulated evidence.

### Eligibility criteria

Studies were eligible for inclusion if they met the following criteria: (1) RCTs; (2) included children ≤18 years of age presenting with acute viral croup; (3) compared low dose (0.15 mg/kg) vs. high dose (0.6 mg/kg) oral dexamethasone; and (4) reported at least one prespecified clinical outcome of interest. Studies were excluded if they: (1) did not report extractable outcome data; (2) included mixed adult and pediatric populations without separable pediatric results; or (3) were non-randomized studies, case reports, reviews, conference abstracts, editorials, or animal studies. No language restrictions were applied during the literature search. Studies published in languages other than English were translated when necessary.

### Search strategy and data extraction

A comprehensive literature search was conducted in PubMed, Embase, and Cochrane Library from database inception through the final search date (November 25, 2025). Controlled vocabulary terms and free-text keywords related to croup, laryngotracheitis, dexamethasone, corticosteroids, pediatric populations, and RCTs were used. Reference lists of included studies and relevant reviews were manually screened to identify additional eligible trials. The complete search strategy is provided in the Supplementary Appendix (Supplementary Table S1).

Search results were imported into Rayyan for deduplication and screening. Three reviewers (A.K., C.L., and T.S.) independently screened titles and abstracts, followed by full-text review of potentially eligible studies. Data extraction was performed using a standardized Microsoft Excel template. Extracted variables included study design, country, sample size, patient demographics, dexamethasone dose, baseline croup severity, and reported outcomes. Discrepancies during screening or extraction were resolved by consensus. Final eligibility and data accuracy were verified by the first author.

### Endpoints and subgroup analyses

Main endpoint was croup severity, as measured by validated croup scoring systems (e.g., Westley croup score), at prespecified time points following dexamethasone administration. Post-treatment croup severity scores were reported at varying time intervals across studies. To enable a clinically meaningful synthesis, we evaluated croup severity at 1 h, 3 h and 4 h after dexamethasone administration, including both change-from-baseline and post-intervention scores, as reported across studies**.** When both were available, post-intervention scores were prioritized.

Additional outcomes included need for hospital admission, requirement for additional interventions (e.g., RE), return to healthcare (emergency department or primary care), and length of stay. Prespecified subgroup analyses to explore potential sources of clinical heterogeneity, including baseline croup severity (mild vs. moderate-to-severe), timing of outcome assessment, and inpatient vs. emergency department-based management, could not be performed as most trials did not report outcomes stratified.

All outcomes reported by the included studies were considered for quantitative synthesis. Meta-analyses were performed only for outcomes for which sufficient data were available across studies. Adverse events were not quantitatively synthesized because they were not reported in the original RCTs.

### Quality assessment

Risk of bias for included RCTs was assessed using the Cochrane Risk of Bias 2.0 tool (RoB-2). Domains evaluated included randomization process, deviations from intended interventions, missing outcome data, outcome measurement, and selective reporting. Two reviewers (A.K. and T.S.) independently assessed risk of bias, with disagreements resolved through discussion.

The certainty of evidence for each outcome was evaluated using the Grading of Recommendations Assessment, Development and Evaluation (GRADE) framework. Evidence certainty was rated as high, moderate, low, or very low based on risk of bias, inconsistency, indirectness, imprecision, and publication bias. GRADE assessments were performed independently by two investigators and finalized by consensus.

### Statistical analysis

Continuous outcomes were pooled as mean differences (MDs) with 95% confidence intervals (CIs), and dichotomous outcomes were pooled as risk ratios (RRs) with 95% CIs. When continuous outcomes were reported as medians with ranges or interquartile ranges, means and standard deviations were estimated using validated methods described by Wan et al. and Luo et al. (12, 13). One study did not report any measure of variance, and therefore, we derived the standard deviation from the highest standard deviation in the same outcomes, following recommended imputation approaches outlined in the Cochrane Collaboration Handbook. This approach has been previously described for handling missing variance data in meta-analyses (10).

Random-effects models were selected *a priori* to account for anticipated clinical and methodological heterogeneity across studies. Between-study heterogeneity was estimated using the restricted maximum likelihood (REML) method and quantified using the *I*^2^ > 25%. Pre-specified subgroup differences were evaluated using *χ*^2^-tests for interaction. Sensitivity analyses were performed using leave-one-out analyses to assess the robustness of pooled estimates. All statistical tests were two-sided, and a *P* value <0.05 was considered statistically significant. All statistical analyses were conducted using R Software (version 2025.09.2 + 418).

TSA was performed for the primary outcomes to evaluate the robustness of the cumulative evidence and to account for the risk of random errors due to sparse data and repeated significance testing. A two-sided type I error (α) of 5% and power (1 − β) of 80% were applied. The required information size was estimated using empirically derived effect size and variance from the included studies, with heterogeneity incorporated using a variance-based correction. Monitoring boundaries were constructed using the O'Brien–Fleming *α*-spending approach, and cumulative Z-curves were generated according to the chronological inclusion of studies, scaled by cumulative sample size. Conventional significance boundaries and trial sequential monitoring boundaries were used to interpret statistical significance and futility. All TSA analyses were performed using TSA software (Copenhagen Trial Unit).

### Sensitivity analysis

A leave-one-out sensitivity analysis was performed to assess the robustness of the pooled estimates. Each study was sequentially excluded, and the meta-analysis was re-run to evaluate the influence of individual studies on the overall effect size and heterogeneity. Changes in pooled estimates, 95% confidence intervals, and I² values were examined to identify any disproportionate impact of a single study on the results.

## Results

### Study selection and characteristics

**Figure 1:**
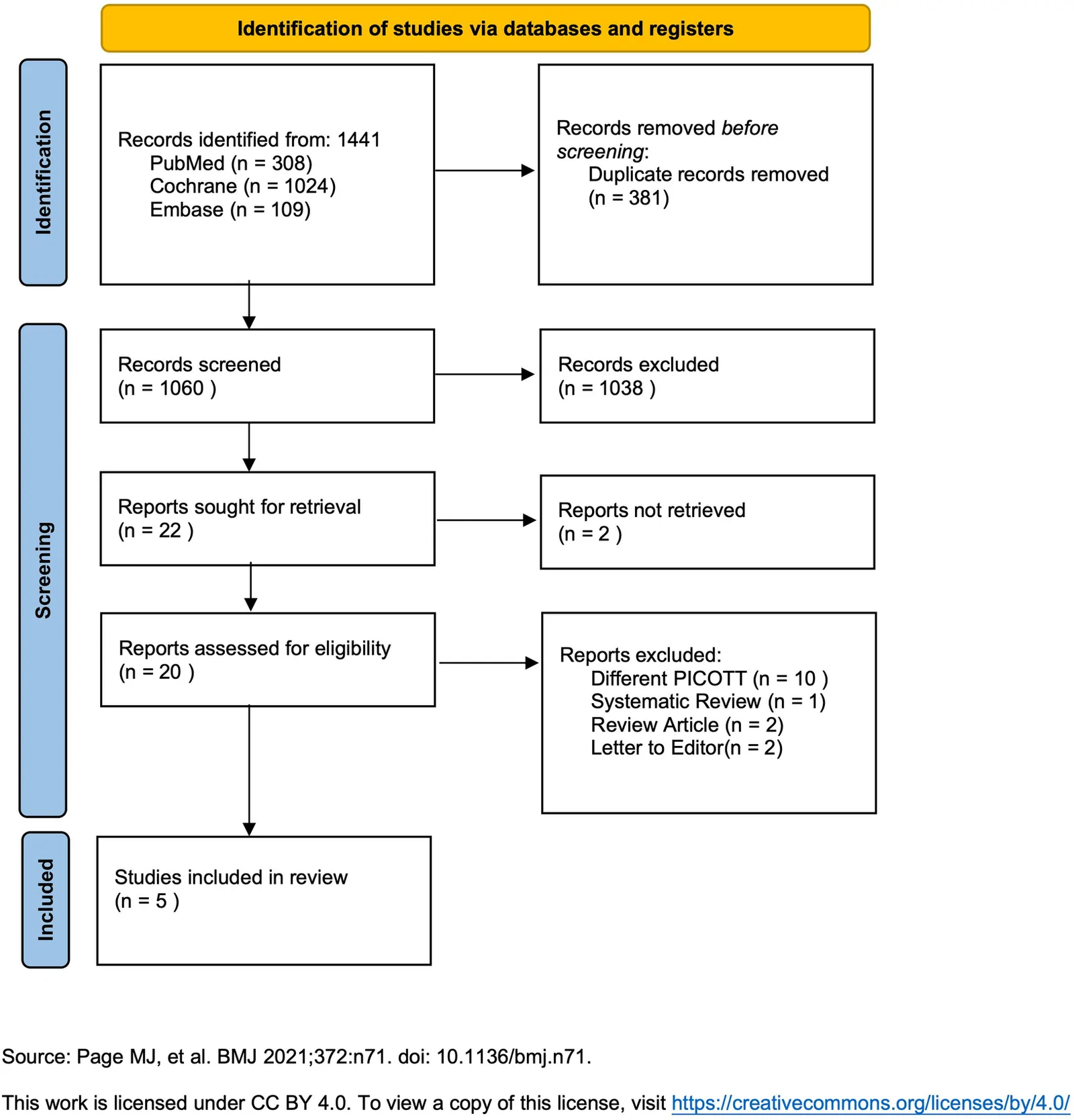
PRISMA 2020 flow diagram for new systematic reviews which included searches of database and registers only.

A total of 1,441 records were identified through database searches. After the removal of duplicate records and title and abstract screening, full texts were sought for 22 reports, of which two could not be retrieved. The remaining 20 full-text articles were assessed for eligibility. Fifteen studies were excluded for the following reasons: different PICOTT criteria (*n* = 10)**,** systematic review (*n* = 1), review articles (*n* = 2), and letters to the editor (*n* = 2) (Supplementary Table S2). Ultimately, five randomized controlled trials were included. Detailed study selection is summarized in Figure 1.

The five included randomized controlled trials comprised 1,058 pediatric patients, of whom 529 (50%) received low-dose (0.15 mg/kg) and 529 (50%) received high-dose (0.6 mg/kg) oral dexamethasone (14–18). The study population consisted predominantly of toddlers, with a mean age of approximately 2–3 years, and included children presenting with croup across a range of severities. Baseline characteristics were generally well balanced between treatment arms within individual trials, including age, sex, baseline croup score, and oxygen saturation when reported. In the study by Fifoot et al. (17), a small proportion of patients had a history of asthma (4 in the low-dose group and 5 in the high-dose group) (17). No other concurrent respiratory infections, such as bronchiolitis or pneumonia, were reported across the included studies. Detailed study characteristics are summarized in Table 1. Additional baseline characteristics are presented in Supplementary Appendix (Supplementary Table S3).

**Table 1:** Baseline characteristics of included randomized clinical trials.

Study	Treatment Arm Participants (n)	Age, months	Male (%)	Initial Croup Score	Previous Croup History (n)^c^	SaO_2_ (%)
Geelhoed (14)	0.15 mg/kg (29)	38 (34)	89%	4	11	97 (1.7)
	0.6 mg/kg (31)	42 (27)	80%	3.7	11	96.6 (1.8)
Alshehr (15)	0.15 mg/kg (36)	13.6 (7.3)	55%	5 (3–6)^b^	5	90 (6)
	0.6 mg/kg (36)	13.6 (7.3)	52%	4.5 (3–6)^b^	4	90 (4)
Chub-Uppakarn (16)	0.15 mg/kg (20)	16.9 (2.0)	55%	4.26 (0.22)	10	96.6 (0.7)
	0.6 mg/kg (21)	18.8 (2.6)	86%	4.60 (0.25)	9.5	96.6 (0.4)
Fifoot (17)	0.15 mg/kg (34)	1.53 (1.31)^a^	65%	2.71 (0.84)	10	NR
	0.6 mg/kg (31)	1.74 (1.61)^a^	68%	2.81 (0.87)	11	NR
Parker (18)	0.15 mg/kg (410)	30.5 (16.3)	62%	1.51 (1.41)	NR	NR
	0.6 mg/kg (410)	29.2 (17.3)	61%	1.4 (1.4)	NR	NR

a Age is presented in years.

b Values are presented as median (IQR).

c Previous croup history indicates the number of participants with a documented prior episode of croup. Unless otherwise indicated, values are presented as mean (SD). Initial croup score refers to the Westley croup score.

SaO_2_ indicates oxygen saturation; NR, not reported.

### Pooled analysis

**Figure 2:**
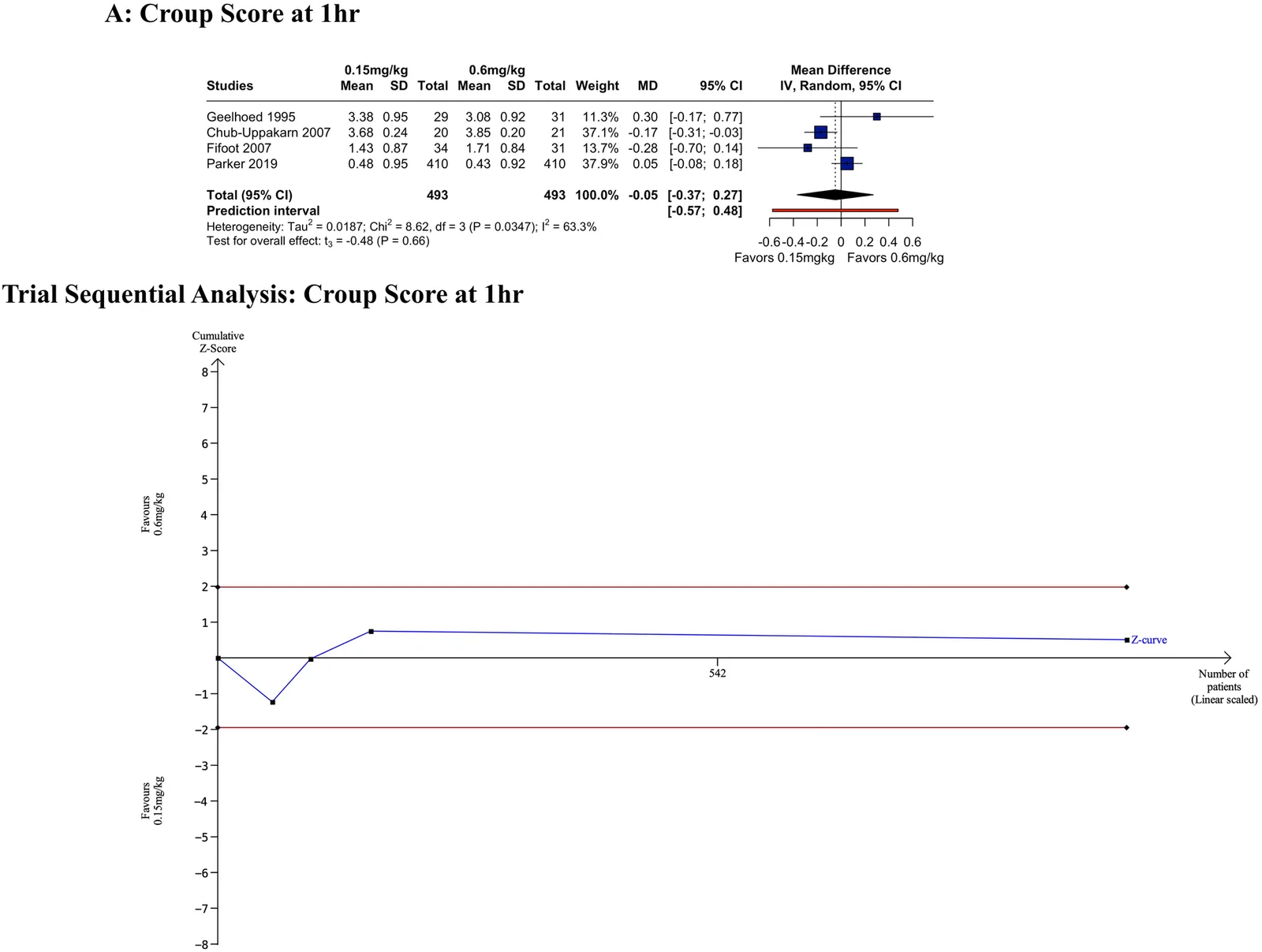

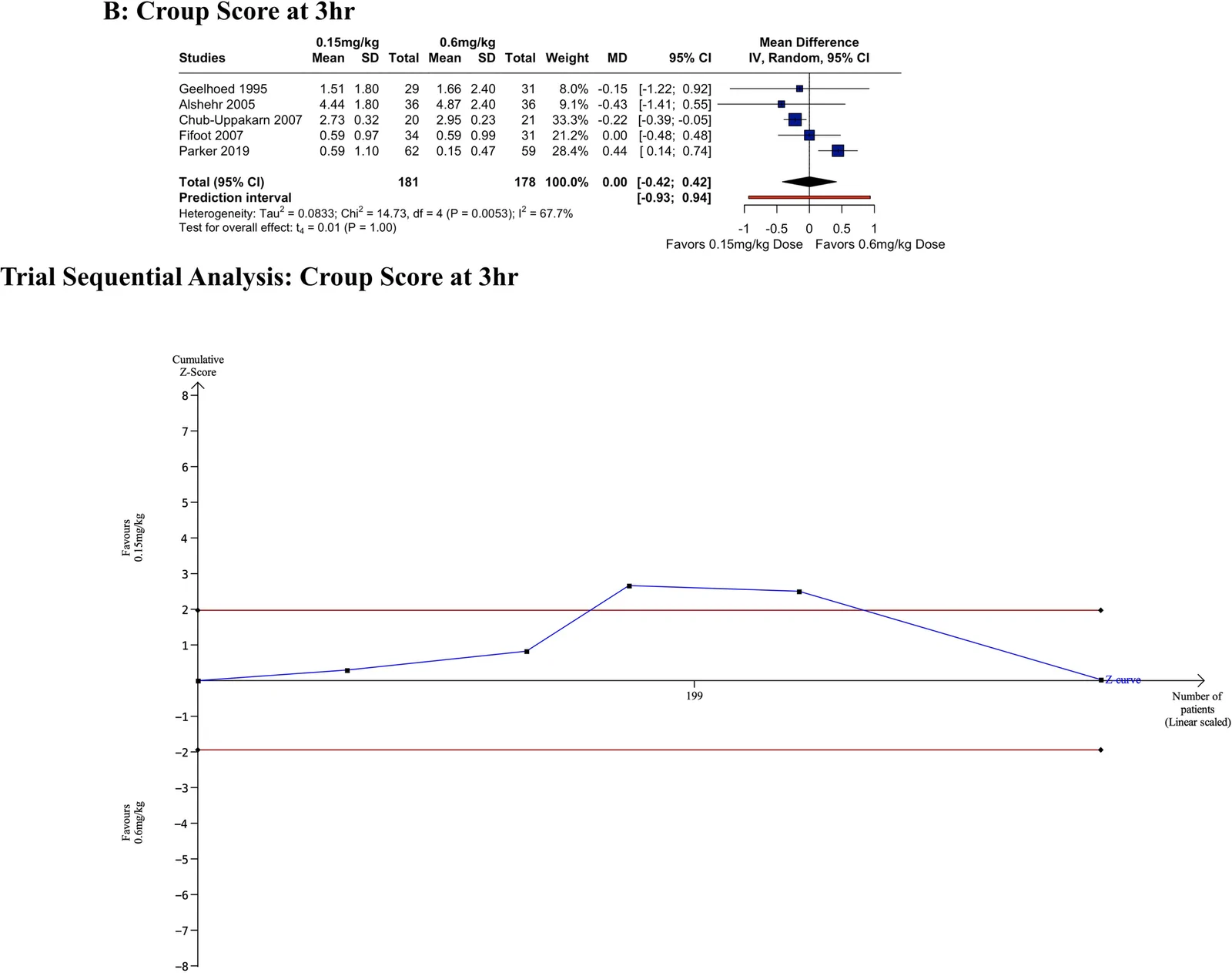

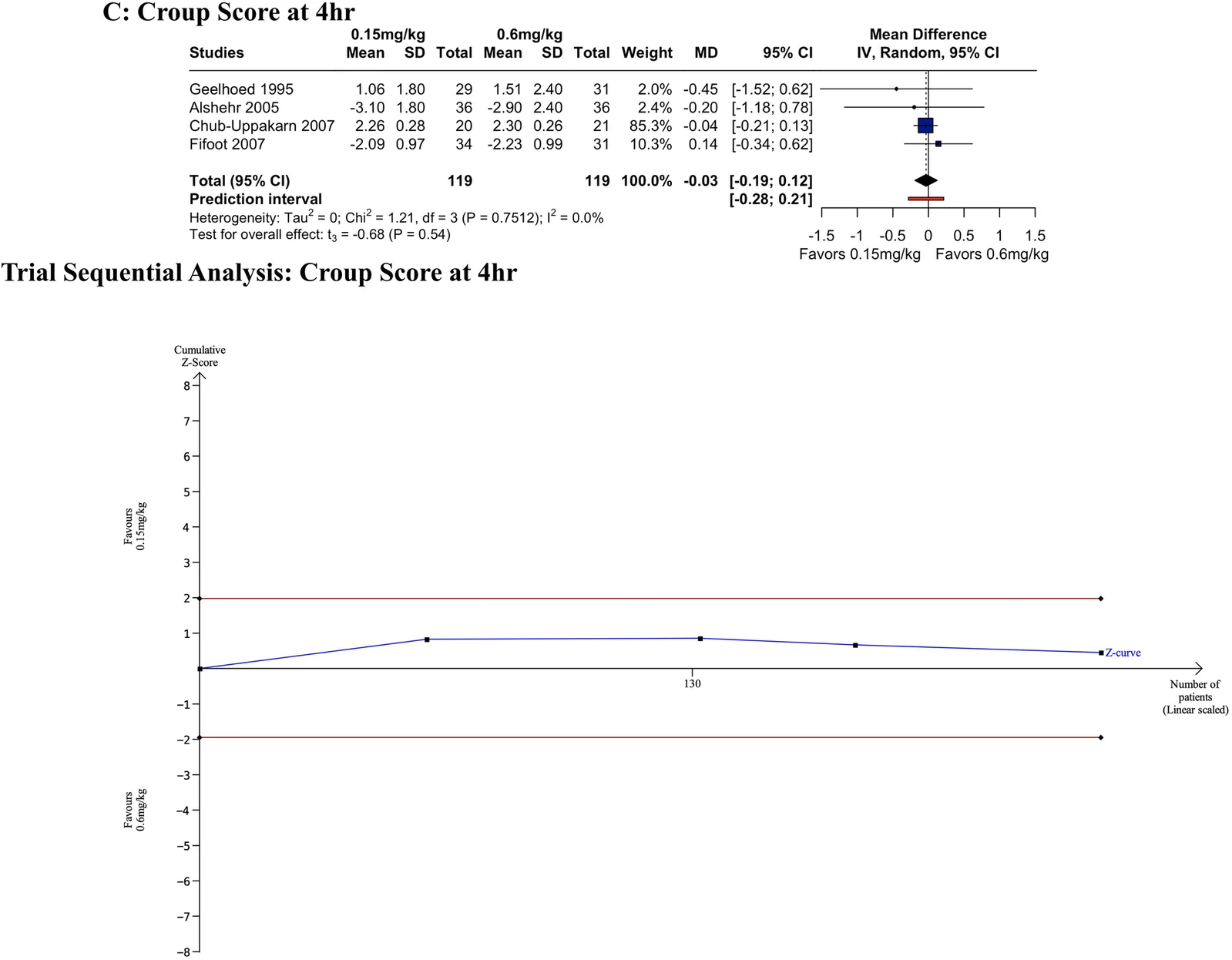
**(A)** Croup score at 1 hr. Trial sequential analysis: croup score at 1 hr. **(B)** Croup score at 3 hr. Trial sequential analysis: croup score at 3 hr. **(C)** Croup score at 4 hr. Trial sequential analysis: croup score at 4 hr.

Pooled analyses of croup severity scores at 1 h, 3 h, and 4 h demonstrated no statistically significant differences between low-dose (0.15 mg/kg) and high-dose (0.6 mg/kg) dexamethasone. At 1 h, four trials including 493 children showed no significant difference between groups (MD −0.05 points; 95% CI −0.37 to 0.27; *P* = 0.66; *I*^2^ = 63.3%; Figure 2A). At 3 h, five trials involving 359 children similarly demonstrated no significant difference (MD 0.00 points; 95% CI −0.42 to 0.42; *P* = 1.00; *I*^2^ = 67.7%; Figure 2B). At 4 h, four trials including 238 children showed comparable outcomes (MD −0.03 points; 95% CI −0.19 to 0.12; *P* = 0.54; *I*^2^ = 0%; Figure 2C). TSA for all three time points showed that the cumulative Z-curves did not cross the conventional significance boundaries or the monitoring boundaries for benefit or harm. Yet, the required information size was not reached for any time point.

**Figure 3:**
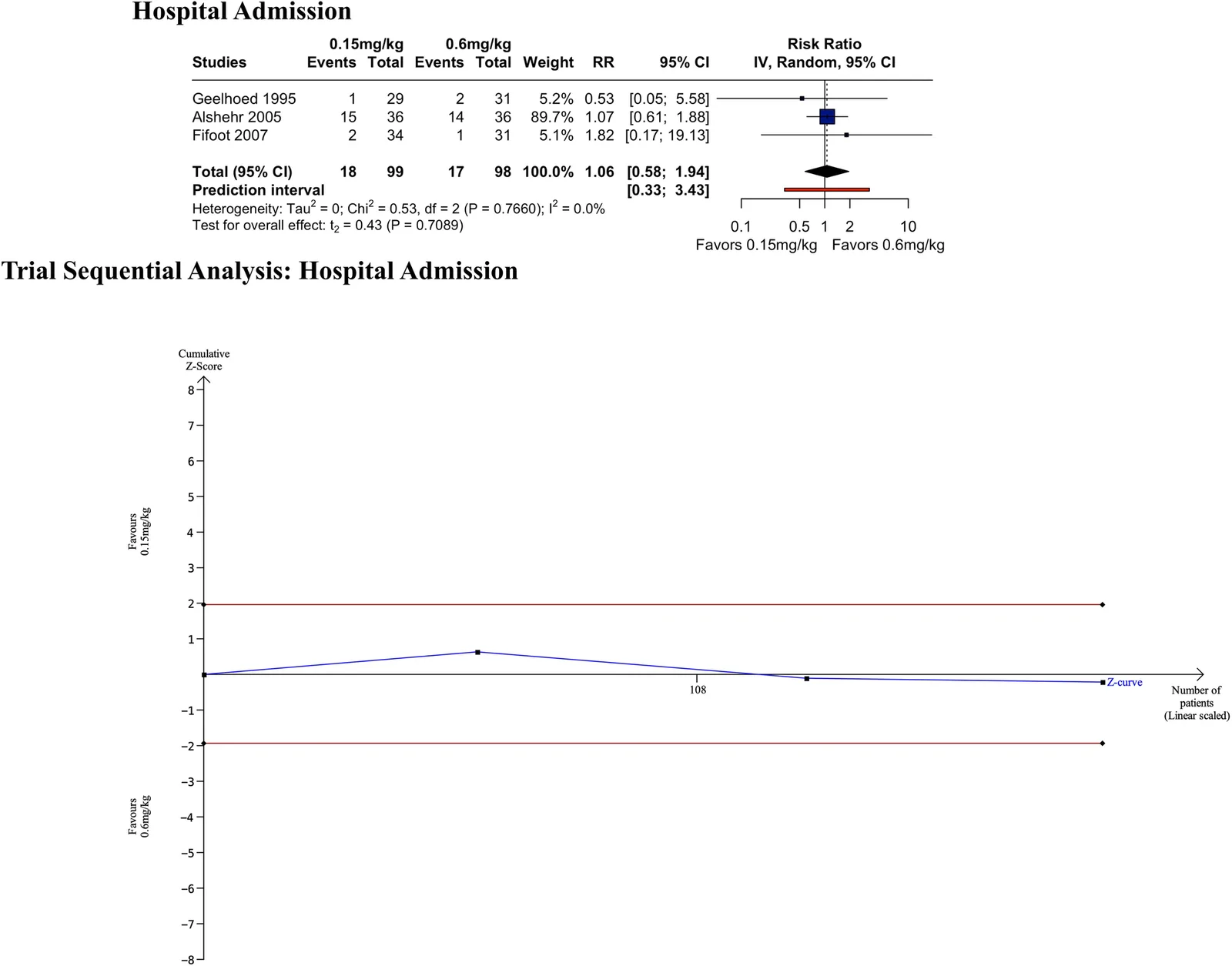
Hospital admission. Trial sequential analysis: hospital admission.

Additionally, there was no significant difference between low-dose and high-dose dexamethasone in hospital admission rates (RR 1.06; 95% CI 0.58 to 1.94; *P* = 0.70; *I*^2^ = 0%; three trials; Figure 3). TSA showed that the cumulative Z-curve did not cross the conventional significance boundaries or the monitoring boundaries for benefit or harm, and the required information size was not reached (Figure 3).

**Figure 4:**
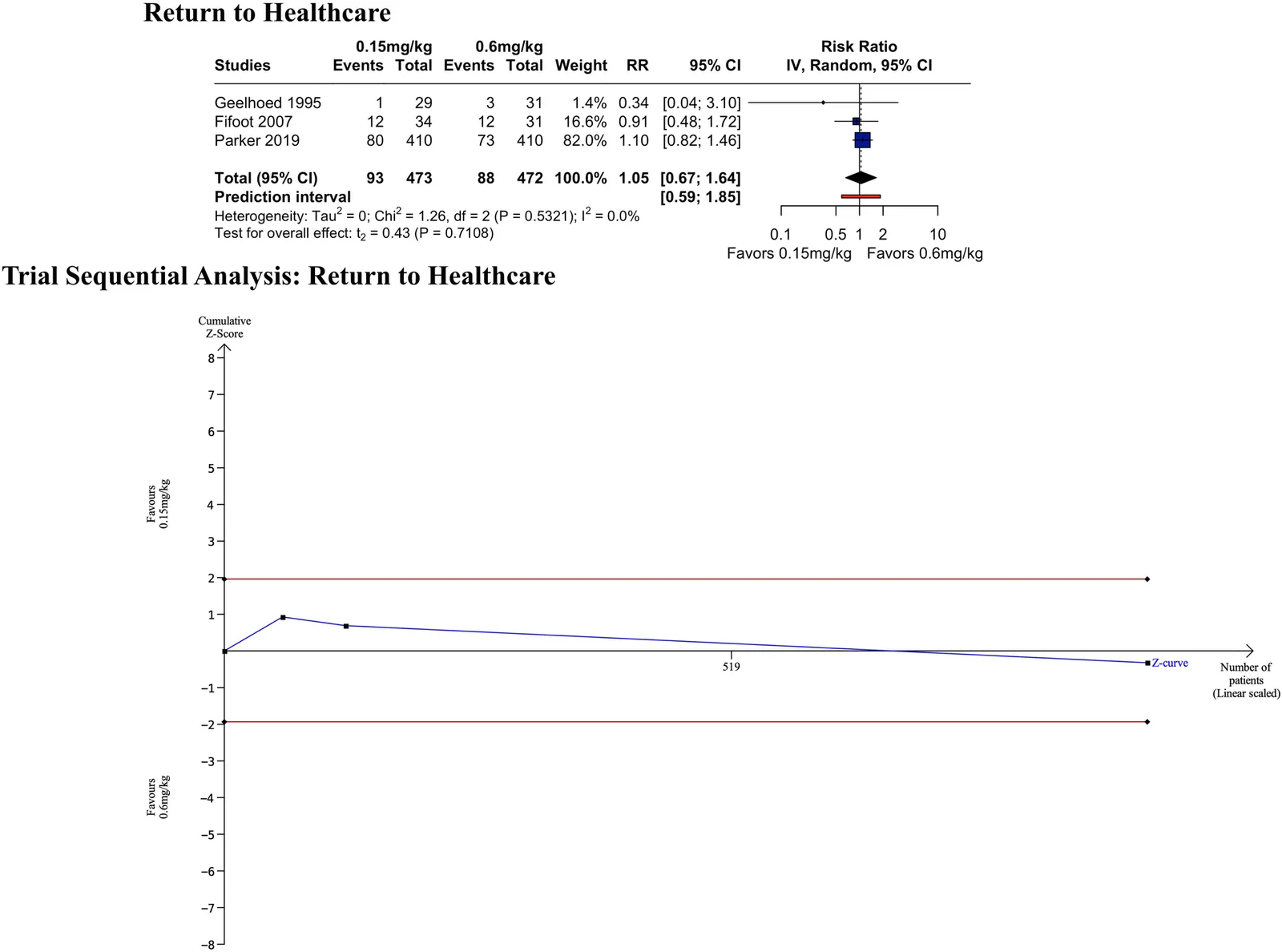
Return to healthcare. Trial sequential analysis: return to healthcare.

Similarly, there was no significant difference in return to healthcare visits (RR 1.05; 95% CI 0.67 to 1.64; *P* = 0.71; *I*^2^ = 0%; three trials; Figure 4). TSA for this outcome also demonstrated that the cumulative Z-curve remained within the monitoring boundaries and failed to reach the required information size (Figure 4). In Parker et al., 80 children in the low-dose group and 73 in the standard-dose group re-presented for medical review; ED re-attendance occurred in 24 and 36 children, respectively, while General Practitioner (GP) re-attendance occurred in 44 and 36 children, respectively.

**Figure 5:**
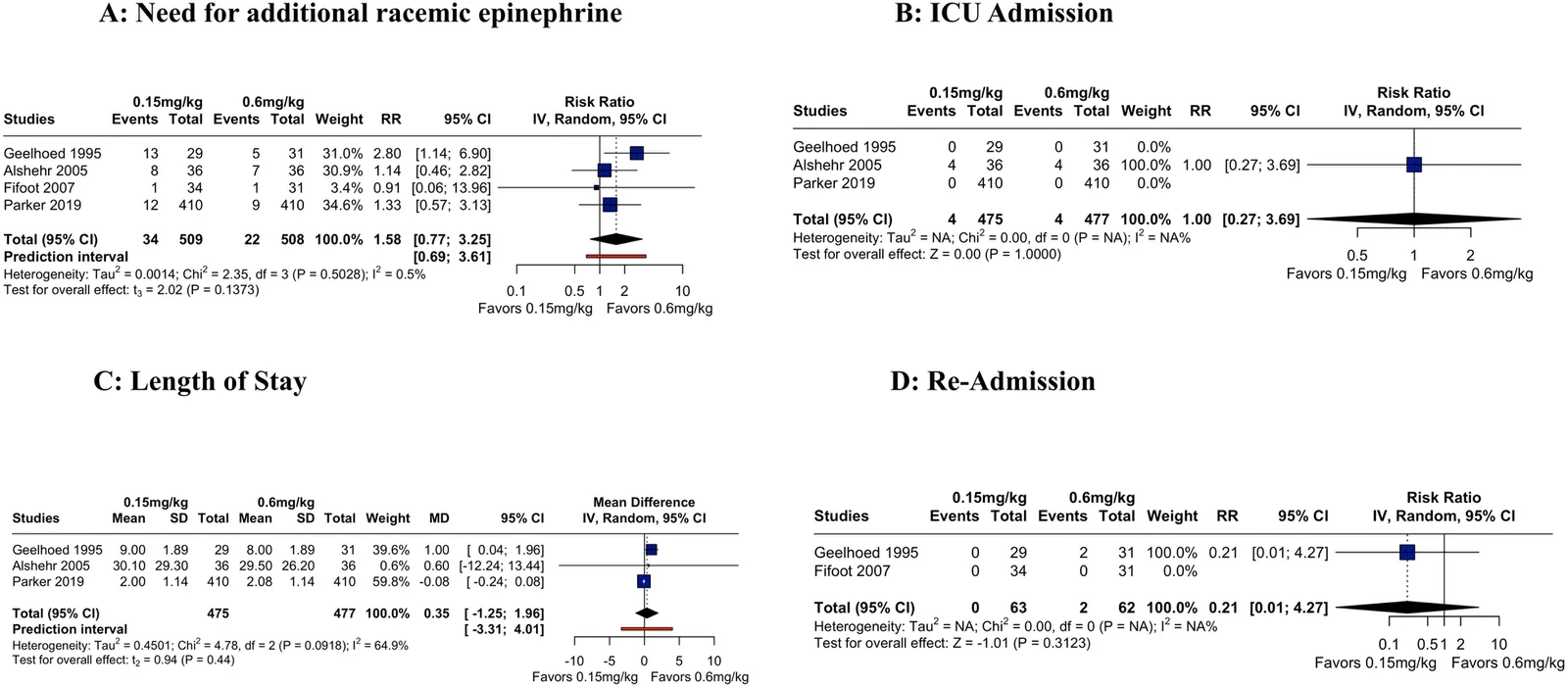
**(A)** Need for additional racemic epinephrine. **(B)** ICU admission. **(C)** Length of stay. **(D)** Re-admission.

The need for additional racemic epinephrine (RR 1.58; 95% CI 0.77 to 3.25; *P* = 0.14; *I*^2^ = 0.5%; four trials; Figure 5A), ICU admission (RR 1.00; 95% CI 0.25 to 3.69; *P* = 1.00; 2 trials; Figure 5B), length of stay (MD 0.35 h; 95% CI −1.25 to 1.96; *P* = 0.44; *I*^2^ = 64.9%; three trials; Figure 5C), readmission with croup (RR 0.21; 95% CI 0.01 to 4.27; *P* = 0.31; Figure 5D), and adverse events (RR 0.63; 95% CI 0.00 to 4,745.57; *P* = 0.63) were also comparable between groups.

It is important to note that Geelhoed et al. consisted of two sequential randomized comparisons (Trial A 0.6 vs. 0.3 mg/kg and Trial B 0.3 vs. 0.15 mg/kg). To address the predefined comparison of low-dose (0.15 mg/kg) vs. high-dose (0.6 mg/kg), an indirect comparison was performed using data from these two trials. Although these groups were not randomized within the same trial, baseline characteristics were similar across all trials.

### Sensitivity analysis

Leave-one-out sensitivity analyses were performed for all outcomes, including hospital admission, return visits, length of stay, need for additional RE, croup scores, and adverse events. Sequential omission of individual studies did not significantly alter pooled effect estimates or overall conclusions. While exclusion of the largest trial influenced confidence interval width for certain outcomes, direction and statistical significance remained unchanged across analyses (Supplementary Figures S1–S4). Given that the comparison between 0.6 mg/kg and 0.15 mg/kg in the Geelhoed study was indirect, we performed a sensitivity analysis restricted to the direct within-trial comparison (0.6 mg/kg vs. 0.3 mg/kg) for croup severity score. This analysis demonstrated no significant difference between groups (MD −0.03 points; 95% CI −0.14 to 0.09; *P* = 0.53; *I*^2^ = 0%; Supplementary Figure S05). These findings were consistent with the primary analysis, with minimal change in effect estimates and confidence intervals.

### Risk of bias and certainty of evidence

Overall risk of bias was judged as low in four studies (14–16, 18) and some concerns in one study (17) related to lack of blinding and incomplete reporting of allocation concealment. Attrition and selective outcome reporting were minimal (Supplementary Table S4).

Using the GRADE framework, certainty of evidence was rated as moderate for hospital admission, need for additional RE, and adverse events, downgraded primarily for imprecision. Certainty was low for return visits and length of stay due to sparse events, wide confidence intervals, and heterogeneity. Moreover, certainty was low to moderate for croup severity scores, reflecting variability in measurement timing and reporting (Supplementary Table S5).

Publication bias was not formally assessed because fewer than 10 studies were available for each outcome. In accordance with Cochrane recommendations, funnel plots and statistical tests for small-study effects (e.g., Egger's regression test) were not performed, as these methods have limited power and may produce misleading results when fewer than 10 studies are included.

## Discussion

In this systematic review and meta-analysis of five RCTs comparing low-dose (0.15 mg/kg) vs. high-dose (0.6 mg/kg) oral dexamethasone in children with croup, we found no differences across all outcomes evaluated, including recurrent symptoms, return to healthcare visits, hospital admission, length of stay, need for rescue epinephrine, and post-treatment croup severity. Our results are consistent with conclusions from prior evidence syntheses suggesting similar clinical effectiveness between the two dosing strategies. However, TSA demonstrated that the cumulative evidence remains underpowered, as the required information size was not reached, and monitoring boundaries for benefit, harm, or futility were not crossed. Importantly, the absence of statistically significant differences should not be interpreted as evidence of equivalence. Rather, the currently available evidence suggests that additional adequately powered randomized trials remain necessary before definitive conclusions can be drawn. Interpretation of the pooled differences in Westley Croup Score should also consider clinical significance. To date, a validated minimal clinically important difference (MCID) for the Westley Croup Score has not been established. Consequently, our conclusions regarding the absence of clinically meaningful differences are based on the small magnitude of the pooled mean differences, the corresponding confidence intervals, and the consistency of findings across other clinically relevant outcomes, rather than comparison with a predefined MCID threshold.

Our meta-analysis differs from prior reviews, including the Cochrane review, particularly in evaluating TSA. Whereas previous reviews evaluated dexamethasone within broader comparisons of corticosteroid formulations, doses, and routes of administration, we restricted inclusion to head-to-head RCTs comparing oral dexamethasone 0.15 mg/kg and 0.6 mg/kg. In addition, ours is the first meta-analysis to incorporate TSA into this clinical question, providing an assessment of whether the accumulated evidence is sufficiently robust to support definitive dosing recommendations. Notably, TSA demonstrated that despite the absence of significant differences across outcomes, the available evidence remains insufficient to establish equivalence with confidence.

Earlier narrative and systematic reviews identified multiple randomized trials reporting no significant differences in croup score reduction across dexamethasone doses; however, the available evidence was insufficient to support definitive dose reduction recommendations (5, 19). In a subsequent risk–benefit assessment, Yates and Doll (20) also concluded that a 0.15 mg/kg dose of dexamethasone was likely to produce clinical effects comparable to the standard 0.6 mg/kg dose. Taken together, our findings build upon this existing literature by providing focused, methodologically rigorous evidence to support dose equivalence. Although conventional meta-analysis suggests similar effectiveness, TSA indicates that the cumulative evidence remains inconclusive because the required information size has not yet been achieved.

Current clinical guidelines recommend corticosteroids for croup across all severity levels, with dexamethasone 0.6 mg/kg widely used despite uncertainty regarding the optimal dose (3, 6, 19). Our findings provide reassurance that currently available evidence does not demonstrate clinically meaningful inferiority of the 0.15 mg/kg dose. However, because TSA showed that the available evidence remains underpowered, these findings should be interpreted cautiously and should not be considered sufficient to justify definitive changes to guideline recommendations. If future adequately powered trials confirm equivalent efficacy, lower-dose dexamethasone could reduce unnecessary corticosteroid exposure while supporting medication stewardship and high-value pediatric care. This approach aligns with the “Minimum Effective Dose” principle and the Choosing Wisely initiative, while also addressing safety concerns given evidence that even brief corticosteroid bursts in children carry measurable risks (21, 22).

The comparable effectiveness of low- and high-dose dexamethasone can be explained by glucocorticoid pharmacology and croup pathophysiology (23). While genomic effects regulate protein synthesis over several hours, rapid non-genomic effects, such as vasoconstriction and reduction of mucosal edema, occur within minutes and play a key role in early clinical improvement (23–27). These non-genomic mechanisms reach a saturation point at relatively low drug concentrations, creating a “ceiling effect” at approximately the 0.15 mg/kg dose (28). Beyond this threshold, higher doses such as 0.6 mg/kg may occupy additional receptors but do not produce additional anti-inflammatory benefit. This suggests that once maximal airway response is achieved, further dose escalation does not translate into improved clinical outcomes.

This meta-analysis has limitations. Most importantly, TSA demonstrated that the cumulative evidence remains underpowered, limiting our ability to establish definitive equivalence between dosing strategies. Additionally, a substantial proportion of the pooled sample (>80%) was derived from a single large trial (Parker et al.), which included patients with relatively lower baseline croup severity compared with other studies. Although sensitivity analyses were performed to assess the impact of this study on the overall findings, the results may still be influenced by its weighting in the pooled estimates. Another limitation is that the included trials were published between 1995 and 2019. During this period, changes in emergency department practice, supportive care, discharge criteria, and corticosteroid prescribing may have introduced clinical heterogeneity that could not be fully accounted for despite consistent eligibility criteria.

However, we sought to evaluate dose equivalence in children with more severe croup (baseline croup score >5), only three trials included this population, and outcome reporting was insufficiently standardized to allow a meaningful subgroup synthesis. Therefore, the lack of significant differences across outcomes should be interpreted cautiously. As a result, the generalizability of low-dose dexamethasone to higher-severity cases remains uncertain. Heterogeneity observed in some outcomes likely reflects differences in baseline disease severity, which directly influence hospitalization duration, discharge timing, and return to healthcare visits. Future randomized trials should focus on moderate-to-severe croup, with stratification by baseline severity and direct dose-comparison designs to better define optimal dosing in higher-risk populations. In addition, future studies should incorporate patient-centered measures such as caregiver satisfaction, medication acceptability, and treatment adherence, alongside systematic assessment of safety outcomes. Economic evaluations comparing dosing strategies would further inform guideline development and support efficient use of healthcare resources. Our findings support that additional RCT evidence remains necessary before definitive recommendations regarding optimal dosing can be made.

## Conclusion

In this systematic review and meta-analysis of five RCTs, a low dose of oral dexamethasone (0.15 mg/kg) showed no clinically meaningful differences compared with the traditional 0.6 mg/kg dose for the management of mild to moderate croup. However, TSA indicated that the cumulative evidence remains underpowered. These findings suggest that 0.15 mg/kg dexamethasone is a promising lower-dose strategy, but they do not yet support definitive de-implementation of the 0.6 mg/kg regimen across all clinical settings. Future adequately powered trials, particularly among children with moderate-to-severe croup, are needed to confirm whether the lower dose can be routinely adopted as the minimum effective dose.

## Data Availability

The datasets presented in this study can be found in online repositories. The names of the repository/repositories and accession number(s) can be found in the article/Supplementary Material.
