# Supplementary material for: Low-dose (0.15 mg/kg) versus high-dose (0.6 mg/kg) dexamethasone for pediatric croup: a meta-analysis with trial sequential analysis of RCTs
Source: Front Pediatr. 2026 Aug 21;14:1925971. doi: 10.3389/fped.2026.1925971 (PMC13541718; doi:10.3389/fped.2026.1925971)
Supplement: Supplementary file 1 [file Supplementaryfile1.docx]

**Supplemental Material**

**Content**

Supplementary Table-1: Full search string….………………………………………3

Supplementary Table-2 Reasons for exclusion after full-text review...……………..4

Supplementary Table-3: Additional baseline Characteristics……………………….6

Supplementary Figures: Sensitivity Analysis………………………………………..7

Supplementary Figures: Sensitivity Analysis with Geelhoed Trial A……………….9

Supplementary Table-4: Risk of Bais Assessment…………………………………..11

Supplementary Table-5: Grading of Recommendations…………………………….12

**Supplementary Table- 1. Full Search Strategy**

| Database | Search Strategy |
| --- | --- |
| PubMed | (Croup[Mesh] OR "Parainfluenza Virus 2, Human"[Mesh]) AND ( Steroids [Mesh] OR Dexamethasone[Mesh] OR Glucocorticoids[Mesh] OR Adrenal Cortex Hormones[Mesh] OR Hydrocortisone[Mesh] OR Prednisolone[Mesh] OR Prednisone[Mesh] OR Methylprednisolone[Mesh] OR Betamethasone[Mesh] OR Triamcinolone[Mesh] OR steroid*[tiab] OR corticosteroid*[tiab] OR glucocorticoid*[tiab] OR "cortico steroid*"[tiab] OR "corticosteroid therapy"[tiab] OR "systemic steroid*"[tiab] OR "oral steroid*"[tiab] OR dexamethason*[tiab] OR hydrocortison*[tiab] OR methylprednisolon*[tiab] OR prednisolon*[tiab] OR prednison*[tiab] OR betamethason*[tiab] OR triamcinolon*[tiab]) AND (Infant[Mesh] OR Child[Mesh] OR Adolescent[Mesh] OR infant*[tiab] OR neonate*[tiab] OR newborn*[tiab] OR toddler*[tiab] OR child[tiab] OR children[tiab] OR kid[tiab] OR schoolchild*[tiab] OR pediatric*[tiab]OR paediatric*[tiab] OR adolescent*[tiab] OR youth*[tiab] OR minors[tiab] OR juvenile*[tiab] OR preschool*[tiab] OR "pre-school"[tiab] OR "school-aged"[tiab]) |
| Embase | ('croup' OR 'parainfluenza virus 2, human') AND ('steroids' OR 'dexamethasone' OR 'glucocorticoids' OR 'adrenal cortex hormones' OR 'hydrocortisone' OR 'prednisolone' OR 'prednisone' OR 'methylprednisolone' OR 'betamethasone' OR 'triamcinolone' OR 'steroid*' OR 'corticosteroid*' OR 'glucocorticoid*' OR 'cortico steroid*' OR 'corticosteroid therapy' OR 'systemic steroid*' OR 'oral steroid*' OR 'dexamethason*' OR 'hydrocortison*' OR 'methylprednisolon*' OR 'prednisolon*' OR 'prednisone' OR 'betamethason*' OR 'triamcinolon*') AND ('infant' OR 'child' OR 'adolescent' OR 'infant*' OR 'neonate*' OR 'newborn*' OR 'toddler*' OR 'child' OR 'children' OR 'kid' OR 'schoolchild' OR 'pediatric*' OR 'paediatric*' OR 'adolescent*' OR 'youth*' OR 'minors' OR 'juvenile*' OR 'preschool*' OR 'pre-school') |
| Cochrane | (Croup OR "Parainfluenza Virus 2, Human") AND (Steroids OR Dexamethasone OR Glucocorticoids OR Adrenal Cortex Hormones OR Hydrocortisone OR Prednisolone OR Prednisone OR Methylprednisolone OR Betamethasone OR Triamcinolone) AND (Infant OR Child OR Adolescent OR neonate OR newborn OR toddler OR child OR children OR kid OR schoolchild OR pediatric OR paediatric OR adolescent OR youth OR minors OR juvenile OR preschool OR "pre-school" OR "school-aged") |

**Supplementary Table-2. Full Text Exclusion**

| **Reasons** | **Articles** |
| --- | --- |
| Wrong PICOTT | 1- Asif, A., Tayyab, A., Qazi, S., Zulfqar, R., Hussain, I., & Mumtaz, H. (2023). Comparison between single-dose oral prednisolone and oral dexamethasone in the treatment of croup: a randomized-controlled trial. *Annals of Medicine and Surgery*, *85*(5), 1379-1384. 2- Geelhoed, G. C., & Macdonald, W. B. (1995). Oral and inhaled steroids in croup: a randomized, placebo-controlled trial. *Pediatric pulmonology*, *20*(6), 355–361. <https://doi.org/10.1002/ppul.1950200604> 3- Dobrovoljac, M., & Geelhoed, G. C. (2012). How fast does oral dexamethasone work in mild to moderately severe croup? A randomized double‐blinded clinical trial. *Emergency Medicine Australasia*, *24*(1), 79-85. 4- James, J. A. (1969). Dexamethasone in croup: a controlled study. *American Journal of Diseases of Children*, *117*(5), 511-516 5- Leipzig, B., Oski, F. A., Cummings, C. W., Stockman, J. A., & Swender, P. (1979). A prospective randomized study to determine the efficacy of steroids in treatment of croup. *The Journal of pediatrics*, *94*(2), 194-196 6- Eden, A. N., Kaufman, A., & Yu, R. (1967). Corticosteroids and croup: controlled double-blind study. *JAMA*, *200*(5), 403-404 7- Amir, L., Hubermann, H., Halevi, A., Mor, M., Mimouni, M., & Waisman, Y. (2006). Oral betamethasone versus intramuscular dexamethasone for the treatment of mild to moderate viral croup: a prospective, randomized trial. *Pediatric emergency care*, *22*(8), 541-544. 8- Haque, K. N. (1981). Efficacy of dexamethasone in acute laryngotracheobronchitis (croup). *Saudi Medical Journal*, *2*(3), 143-5.   9- Efficacy Of Oral Dexamethasone Versus Parenteral Dexamethasone In The Management Of Mild To Moderate Croup  ClinicalTrials.gov ID NCT06959407   10- Postma, D. S., Jones, R. O., & Pillsbury III, H. C. (1984). Severe hospitalized croup: treatment trends and prognosis. *The Laryngoscope*, *94*(9), 1170-1175 |
| Review Article | 1- Rowe, B. H. (2002). Corticosteroid treatment for acute croup. Annals of emergency medicine, 40(3), 353-355. 2- Geelhoed, G. C. (1996). Croup: new perspectives on corticosteroid therapy. Clinical Immunotherapeutics, 5(4), 260-267 3- Hibberd, O., Chylinska, A. A., Finn, K., Ranaweera, M., Hall, D., & Don't Forget The Bubbles (2024). Use of corticosteroids for croup in children. Archives of disease in childhood. Education and practice edition, 109(6), 306–311. https://doi.org/10.1136/archdischild-2023-326773 |
| Letter to Editor | 1- Klassen, T. P., Feldman, M. E., Watters, L. K., Sutcliffe, T., & Rowe, P. C. (1994). Nebulized budesonide for children with mild-to-moderate croup. New England Journal of Medicine, 331(5), 285-289. 2- Harel, Y., & Levin, D. L. (1995). Aerosolized corticosteroids for croup. The New England Journal of Medicine, 332(6), 400-400. |
| No PDF | 1- Croup Dosing Study ClinicalTrials.gov ID NCT06272383   2- de Benedictis, F. M., & Vaccaro, R. (2000). Il ruolo dei corticosteroidi nella terapia del croup. *RIVISTA ITALIANA DI PEDIATRIA*, *26*(4), 698-703. |

**Supplementary Table-3. Additional Baseline Characteristics**

# Table 3. Additional Baseline Characteristics of Included Studies Comparing Dexamethasone 0.15 mg/kg vs 0.6 mg/kg for Croup

| Characteristic | Geelhoed et al., 1995 | | Alshehri et al., 2005 | | Chub-Uppakarn et al., 2007 | | Fifoot et al., 2007 | | Parker et al., 2019 | |
| --- | --- | --- | --- | --- | --- | --- | --- | --- | --- | --- |
| Study design | RCT | | RCT | | RCT | | RCT | | RCT | |
| Study setting | Emergency department | | Emergency department | | Emergency department | | Emergency department | | NR | |
| Country | Australia | | Saudi Arabia | | Thailand | | Australia | | NR | |
| Population | Children with croup | | Children with croup | | Children with moderate croup | | Children with croup | | NR | |
| I/C | 0.15mg/kg | 0.6mg/kg | 0.15mg/kg | 0.6mg/kg | 0.15mg/kg | 0.6mg/kg | 0.15mg/kg | 0.6mg/kg | 0.15mg/kg | 0.6mg/kg |
| Mean/median age ^*^ | 38(34) | 42(27) | 13.6 (7.3) | 13.6 (7.3) | 16.9(2.0) | 18.8(2.6) | 1.53  (1.31)^*^ | 1.74  (1.61)^*^ | 30.5(16.3) | 29.2(17.3) |
| Male sex, n (%) | 26 | 25 | 20 | 19 | 55% | 86% | 22(65%) | 21(68%) | NR | NR |
| Sample size (n) | 29 | 31 | 36 | 36 | 20 | 21 | 34 | 21 | 410 | 410 |
| Baseline croup  severity | Mild–moderate | Mild–moderate | Moderate (score ≥3) | Moderate (score ≥3) | Moderate (score ≥3) | Moderate (score ≥3) | Mild–moderate | Mild–moderate | Mild | Mild |
| Baseline Westley ^†^  croup score | 4 | 3.7 | 5(3-6)^†^ | 4.5(3-6)^†^ | 4.26(0.22) | 4.60(0.25) | 2.71(0.84) | 2.81(0.87) | 1.5(1.4) | 1.4(1.4) |
| Croup Score  0-1 (n) | NR | NR | NR | NR | NR | NR | NR | NR | 216(52.9%) | 226(55.4%) |
| Croup Score  2-3 (n) | NR | NR | NR | NR | NR | NR | NR | NR | 165(40.4) | 157(38.5) |
| Croup Score  4+ (n) | NR | NR | NR | NR | NR | NR | NR | NR | 27(6.6) | 25(6.1) |
| Previous Croup (n) | 11 | 11 | 5 | 4 | 10 | 9.5 | 10 | 11 | NR | NR |
| Viral/spasmodic) Croup | 6/8 | 12/3 | NR | NR | NR | NR | NR | NR | NR | NR |
| Duration of Symptoms (hr) | 11 (17) | 25(31) | NR | NR | NR | NR | NR | NR | NR | NR |
| Duration of fever (days) | NR | NR | 10(0-24) | 9(0-22) | 2.1(0.2) | 2.3(0.3) | NR | NR | NR | NR |
| Duration of rhinorrhea  (days) | NR | NR | NR | NR | 1.6(0.3) | 1.7(0.4) | NR | NR | NR | NR |
| Duration of hoarseness (days) | NR | NR | NR | NR | 1.2(0.2) | 1.9(0.4) | NR | NR | NR | NR |
| Duration of dyspnea (days) | NR | NR | NR | NR | 1.4(0.2) | 1.5(0.4) | NR | NR | NR | NR |
| Temparture © | 37.4(0.9) | 37.8(1.2) | 27.8(1.1) | 37.6(1.3) | NR | NR | NR | NR | NR | NR |
| Pulse rate (min) | 148(29) | 144(23) | 140.4(22.5) | 136(20.4) | NR | NR | NR | NR | NR | NR |
| Respiratory rate (min) | 33(9) | 33(9) | 39(10) | 36(6) | NR | NR | NR | NR | NR | NR |
| SaO2 in RA (%) | 97%(1.7) | 96.6(1.8) | 90(6) | 90(4) | 96.6(0.7) | 96.6(0.4) | NR | NR | NR | NR |
| Route of dexamethasone | Oral | Oral | Oral | Oral | IV | IV | Oral | Oral | Oral | Oral |
| Single-dose administration | Yes | Yes | Yes | Yes | Yes | Yes | Yes | Yes | Yes | Yes |
| No. of patient with preceding URI (n) | NR | NR | NR | NR | NR | NR | 22(65%) | 25(80%) | NR | NR |
| No. of pt with Asthma | NR | NR | NR | NR | NR | NR | 4(12%) | 5(16%) | NR | NR |
| Prior racemic epinephrine use | NR | NR | 3 | 3 | NR | NR | NR | NR | NR | NR |

*Age is presented in years

^†^ Values are presented as median (IQR)
© Temperature reported in degrees Celsius

Data are presented as mean (SD) unless otherwise specified
Viral/spasmodic croup indicates classification as reported by the original study authors.
Oxygen saturation (SaO) was measured on room air.NR indicates not reported; RCT, randomized controlled trial; I, intervention group (dexamethasone 0.15 mg/kg); C, control group (dexamethasone 0.6 mg/kg); URI, upper respiratory infection; IV, intravenous; RA, room air.

**Supplementary Figure-01: Adverse Events**

**Leave-one-out Analysis**

**Supplementary Figure-2: Croup Score at 1 Hours**

**Supplementary Figure-3: Croup Score at 3Hours**

**Supplementary Figure-4: Croup Score at 4 Hours**

**Supplementary Figure-5: Hospital Admission**

**Supplementary Figure-6: Return to Healthcare**

**Supplementary Figure-7: Need for Additional Racemic Epinephrine**

**Sensitivity Analysis (with Geelhoed Trial A)**

**Supplementary Figure-8: Geelhoed Trial A**

**Supplementary Figure-9: Leave-one-out**

**Supplementary Table-4. Risk of Bias Assessment**

**Risk of bias summary for randomized studies (RoB 2)**

| **Study** | **Bias from randomization process** | **Bias due to deviations from intended interventions** | **Bias due to missing outcome data** | **Bias in measurement of the outcomes** | **Bias in selection of the reported result** | **Overall risk of bias** |
| --- | --- | --- | --- | --- | --- | --- |
| Geelhoed 1995 | Low | Low | Low | Low | Some Concerns | Low |
| Alshehr 2005 | Low | Low | Low | Low | Low | Low |
| Chub- Uppakarn 2007 | Some Concerns | Low | Low | Low | Low | Low |
| Fifoot 2007 | Low | Low | Low | Low | Some Concerns | Some Concerns |
| Parker 2019 | Low | Low | Low | Low | Low | Low |

How to cite RoB 2: Sterne JAC, Savović J, Page MJ, Elbers RG, Blencowe NS, Boutron I, Cates CJ, Cheng H-Y, Corbett MS, Eldridge SM, Hernán MA, Hopewell S, Hróbjartsson A, Junqueira DR, Jüni P, Kirkham JJ, Lasserson T, Li T, McAleenan A, Reeves BC, Shepperd S, Shrier I, Stewart LA, Tilling K, White IR, Whiting PF, Higgins JPT. RoB 2: a revised tool for assessing risk of bias in randomised trials. *BMJ* 2019; **366**: l4898.

**Supplementary Table-5. Grading Recommendations**

**Author(s):** Ayesha Khalid MD, Thaís S. Martins Shehan, MD

**Question:** 0.15mg/kg compared to 0.6mg/kg for Pediatric Croup Patient

**Setting:** ED/Inpatient

**Bibliography:** The Westley croup score is an ordinal clinical severity score ranging from 0 to 17 points. Scores were assessed at 4 hours after dexamethasone administration.

| **Certainty assessment** | | | | | | | **№ of patients** | | **Effect** | | **Certainty** | **Importance** |
| --- | --- | --- | --- | --- | --- | --- | --- | --- | --- | --- | --- | --- |
| **№ of studies** | **Study design** | **Risk of bias** | **Inconsistency** | **Indirectness** | **Imprecision** | **Other considerations** | **0.15mg/kg** | **0.6mg/kg** | **Relative (95% CI)** | **Absolute (95% CI)** |  |  |
| **Croup Score at 4hours (assessed with: Westley Croup Score (0–17 points); Scale from: 0 to 17)** | | | | | | | | | | | | |
| 4 | randomised trials | not serious | not serious | serious^a^ | not serious | none | 119 | 119 | - | MD **0.03 Points lower** (0.19 lower to 0.12 higher) | ⨁⨁⨁◯ Moderate^a^ | CRITICAL |
| **Hospital Admission (assessed with: (n)** | | | | | | | | | | | | |
| 3 | randomised trials | not serious | not serious | serious^a^ | not serious | publication bias strongly suspected^b^ | 18/99 (18.2%) | 17/98 (17.3%) | **RR 1.06** (0.58 to 1.94) | **10 more per 1,000** (from 73 fewer to 163 more) | ⨁⨁◯◯ Low^a,b^ | IMPORTANT |
| **Return to Healthcare (assessed with: (n)** | | | | | | | | | | | | |
| 3 | randomised trials | not serious | not serious | serious^a,b^ | not serious | none | 93/473 (19.7%) | 88/472 (18.6%) | **RR 1.05** (0.67 to 1.64) | **9 more per 1,000** (from 62 fewer to 119 more) | ⨁⨁⨁◯ Moderate^a,b^ | IMPORTANT |
| **Need for Additional Racemic Epinephrine (assessed with: (n)** | | | | | | | | | | | | |
| 4 | randomised trials | not serious | not serious | serious^a^ | serious^c^ | none | 34/509 (6.7%) | 22/508 (4.3%) | **RR 1.58** (0.77 to 3.25) | **25 more per 1,000** (from 10 fewer to 97 more) | ⨁⨁◯◯ Low^a,c^ | IMPORTANT |

**CI:** confidence interval; **MD:** mean difference; **RR:** risk ratio

#### Explanations

a. Indirect comparison between 0.15mg/kg to 0.6mg/kg in One study

b. 2 of 5 included trials did not report the outcome

c. Wide Confidence Interval
